# Availability of calorie information on online menus from chain restaurants in the USA: current prevalence and legal landscape

**DOI:** 10.1017/S1368980023001799

**Published:** 2023-12

**Authors:** Eva Greenthal, Sarah Sorscher, Jennifer L Pomeranz, Sean B Cash

**Affiliations:** 1 Center for Science in the Public Interest, Washington, DC 20005, USA; 2 New York University School of Global Public Health, New York, NY, USA; 3 Gerald J. and Dorothy R. Friedman School of Nutrition Science and Policy, Tufts University, Boston, MA, USA

**Keywords:** Menu labelling, Nutrition policy, Food environment, Online food delivery

## Abstract

**Objective:**

Federal law requires calorie information on chain restaurant menus. We sought to assess the prevalence of calorie disclosures on online menus and determine if the menus are controlled by restaurants subject to US labelling requirements.

**Design:**

Cross-sectional

**Setting:**

Restaurant websites and mobile apps for restaurant located in New York City, Los Angeles, Chicago, and Houston

**Participants:**

US chain restaurants (top seventy-five by number of outlets) and third-party platforms (TPP): Grubhub, Uber Eats, DoorDash

**Results:**

There was at least one calorie disclosure (for at least one food or beverage, in at least one location) on sixty-eight of seventy-two (94 %) menus on restaurant websites or apps, thirty-two of fifty-five (58 %) menus on DoorDash, six of forty-nine (12 %) menus on Grubhub and thirty of fifty-nine (51 %) menus on Uber Eats. There was consistent calorie labelling (all foods and beverages, all locations) on forty-three of seventy-two (60 %) menus on restaurant websites or apps, fifteen of fifty-five (27 %) menus on DoorDash, three of forty-nine (6 %) menus on Grubhub and eleven of fifty-nine (19 %) menus on Uber Eats. Only four restaurant chains consistently labelled calories for all items, in all locations, on all platforms where their menus were found. All three TPP provided restaurants the ability to enter and modify menu items, making the menus subject to US labelling requirements. Only Uber Eats provided guidance to restaurants on entering calorie information.

**Conclusions:**

As consumers increasingly rely on TPP for restaurant ordering, menus on these platforms should include calories in order to promote transparency and nutrition.

Positive energy imbalance, caused by consuming more calories than the body needs, can result in weight gain which is linked to adverse health outcomes including type 2 diabetes, heart disease and certain cancers^(1)^. Between 1970 and 2010, there was an increase in estimated daily average per capita intake from 2054 calories to 2501 calories, and more recent data suggest this has remained steady or even increased^(2,3)^. Rates of diet-related diseases, like diabetes, increased over this period as well^(4)^. To address the public health harms of overconsumption, the US government has adopted initiatives to inform consumers about the calorie content of foods, including requirements for calorie disclosures on restaurant menus^(5)^.

Food from chain restaurants accounts for a substantial proportion of Americans’ daily calories, presenting an important opportunity for calorie reduction. As of 2016, fast food restaurants served more than one in three American adults on a given day^(6)^.

Calorie labelling on restaurant menus promotes transparency and informed consumer decision-making and appears to modestly reduce calories purchased. A meta-analysis of three randomised controlled trials found that including calorie counts on menus reduced calories purchased by forty-seven calories per 600-calorie meal or 8 %^(7)^. A quasi-experimental study found a decrease of sixty calories per transaction following implementation of menu labelling requirements which was only partially attenuated by an increase in calories per transaction in the subsequent year^(8)^.

Prior to 2010, there was no federal requirement for calorie information on restaurant menus. In 2010, the Affordable Care Act amended the Federal Food, Drug, & Cosmetic Act to require chain restaurants or similar retail food establishments with twenty or more locations (i.e. Covered Restaurant Establishments (CRE)) to disclose calorie information next to each ‘standard menu item’ on each menu or menu board, along with ‘a succinct statement concerning suggested daily caloric intake’ and a statement that additional nutrition information is available upon request on the premises of the restaurant^(9)^. Foods that do not comply with nutrition labelling requirements in the Federal Food, Drug, & Cosmetic Act are ‘misbranded’, making the sale of such foods a violation of federal law^(10)^.

FDA issued a final rule detailing how this law must be implemented in 2014, and enforcement began in May 2018^(11,12)^. Despite limited enforcement resources^(13)^, a study examining compliance found that 94 % of the 197 highest grossing CRE had implemented calorie labelling on their printed menus by May 2018^(14)^.

In April 2020, FDA issued a temporary guidance to industry stating that the agency would not enforce menu labelling requirements for ‘the duration of the public health emergency related to COVID-19’^(15)^. The federal public health emergency period expires on May 11, 2023,^(16)^ and FDA has stated that the temporary guidance that paused menu labelling enforcement will expire on November 7, 2023^(17)^.

Americans are increasingly purchasing restaurant food from third-party platforms (TPP) like DoorDash, Uber Eats and Grubhub^(18)^, raising the questions of whether menus from CRE on these platforms currently include calories, and whether these menus are covered under existing regulations. This is particularly important given that online menus and TPP are associated with the so-called ‘food delivery revolution’ which is increasing people’s access to unhealthy meals and may be contributing to increased consumption^(19)^.

In 2021, a coalition of consumer advocacy groups wrote to FDA noting examples of CRE menus on TPP that were missing calorie disclosures and asking the agency to clarify that calorie labelling is required on these menus. The groups argued that in order for menu labelling to have its intended effect on public health, consumers must have easy access to the labelled information wherever they make food selections, including on TPP^(20)^. Moreover, under a reasonable interpretation of the federal regulations, CRE’s menus posted on TPP are covered by federal menu labelling requirements^(11)^. FDA has indicated in a statement to the press that the TPP themselves ‘likely would not meet the definition of a covered establishment under our current requirements and therefore would not be subject to menu labelling requirements’^(21)^. But the agency has yet to respond to the advocates’ request to clarify that menus controlled by CRE are subject to menu labelling requirements when posted on such platforms. FDA rules state that ‘if consumers can order from a covered establishment online, … using a writing of the covered establishment on the Internet as the primary writing from which he or she makes his or her order selection, then the writing on the Internet is a menu for the purposes of [nutrition labelling requirements]’^(11)^. The groups have asked FDA to clarify that if a CRE controls the content of a menu posted on a TPP, the menu would remain covered under the law.

This study includes a cross-sectional analysis of online CRE menus assessing the prevalence with which CRE menus posted to TPP include calorie information and comparing the prevalence of calorie disclosures on TPP to the prevalence of disclosures on ordering platforms through CRE websites. We also conducted an analysis of TPP websites to determine whether CRE control the content of their menus posted to these platforms.

## Methods

### Restaurant menu analysis

To examine calorie disclosure on online CRE menus, we obtained a list of the top chain restaurants by 2020 revenue from Nation’s Restaurant News^(22)^. Between June and September 2022, we examined menus from the top seventy-five by number of outlets in four online locations or ‘channels’: DoorDash, Uber Eats and Grubhub (the TPP, which collectively accounted for 96 % of meal delivery sales in May 2022) and CRE websites^(23)^. We pre-registered our study protocol with AsPredicted.org (#101399).

Three coders used iPhones to review the menus. They downloaded the three TPP’ apps and viewed restaurant websites in Safari. If there was no option to order through Safari, coders downloaded the restaurant’s app. For both the CRE’ websites and the three TPP, coders extracted and recorded the following attributes in Microsoft Excel: presence of calories for the first three eligible food and first three eligible beverage items listed on the app or website page; legibility and accessibility of calorie disclosures; and presence of the two disclosures required on CRE menus under FDA regulations.

To assess presence of calorie disclosures, coders took screenshots of the first place a menu item was listed on the app or website and subsequent screenshots after clicking on the item. They followed prompts either until they either saw a calorie disclosure or until the item could be added to their ‘order’.

Definitions of accessibility and legibility were adapted from those used in a previous online labelling study^(24)^. Disclosures were considered fully legible if they could be read without zooming or hovering and were not blurry. Disclosures were considered easily accessible if they could be viewed on the first page where the food was listed, without clicking or scrolling.

These two required disclosures were ‘2000 calories a day is used for general nutrition advice, but calorie needs vary’ (2000 calorie disclosure) and ‘Additional nutrition information available upon request’ (Additional Information Disclosure)^(25)^. Variations of the Additional Information Disclosure, such as ‘Full nutrition facts available here’ with a link to the CRE’s website, were counted as Additional Information Disclosures.

Combination meals, promotions, temporary items (e.g. ‘picked for you’ or ‘limited time offer’) and alcoholic beverages were not eligible for coding because these items may be exempt from menu labelling requirements^(26)^.

Prior to data collection, we conducted a pilot to test the assumption that if the menu from one location of a CRE included calories on a given channel, the menus from all other locations would also include calories on that channel. In this pilot, we found variation across locations for more than 10 % of the CRE/TPP combinations. Therefore, our full protocol examined menus from each CRE on each channel from four different restaurant locations (New York City, Los Angeles, Chicago and Houston). When prompted to enter an address for pickup or delivery, coders entered the address of each city’s City Hall.

A fourth coder double-coded menus from a random selection of 10 % of the restaurants in our sample to assess coding accuracy; inter-rater reliability was 99 %.

We produced descriptive statistics (both unweighted and weighted by number of outlets) and used a chi-squared test for independence and Bonferroni-adjusted comparisons to assess differences in prevalence of calorie disclosures across channels and locations^(27)^. We also calculated Pearson’s r coefficient to examine the correlation between calorie disclosure on TPP and number of outlets. Analyses were conducted with Microsoft Excel Version 2301 and Stata 17^(28)^. Proportions were weighted by dividing the number of outlets among CRE with a given attribute by the total number of outlets among all CRE with a menu on that channel. In the text, we primarily report unweighted proportions, except where there are noteworthy differences between the weighted and unweighted.

### Control of third-party platform websites

To assess whether CRE can control the content of their menus on TPP, a research assistant searched the Grubhub, DoorDash and Uber Eats company websites in September 2022 for instructions explaining how restaurants can post menus on their platforms, attempted to post a hypothetical menu on each TPP and called each TPP’s customer service line using a standardised script. The research assistant collected information including whether and how restaurants can post and update menus; whether and how TPP allow restaurants to include calorie information, 2000 calorie disclosures, and Additional Information Disclosures on menus; whether TPP provide information to restaurants about menu labelling requirements; and whether TPP charged a premium to CRE for posting nutrition information.

## Results

### Restaurant menus

Our sample included seventy-five CRE with a combined total of 183 224 outlets across the USA in 2020 (range = 460–23 801 outlets per CRE). Thirty-eight of the seventy-five CRE (53 %) had less than 1000 outlets each and accounted for 14 % of total outlets, thirty-four (47 %) had between 1000–10 000 outlets each and accounted for 57 % of total outlets, and three (4 %) – Starbucks, Subway and McDonald’s – had over 10 000 outlets each and accounted for 29 % of total outlets (Table 1). The most common restaurant types in our sample were limited-service burger (18 %, unweighted), pizza (14 %), beverage/snack (13 %) and casual dining (13 %). All but three CRE (Waffle House, Bojangles, Tim Hortons), or 96 %, allowed customers to order through the restaurant’s own website or app in at least one of the four cities. Sixty CRE (83 %) had menus in at least one city on at least one TPP, including fifty-five (73 %) with menus on DoorDash, forty-nine (65 %) with menus on Grubhub and fifty-nine (79 %) with menus on Uber Eats. Fifty-six CRE (75 %) had menus available on at least one channel in New York City, sixty (80 %) in Los Angeles, sixty-six (88 %) in Chicago and sixty-six (88 %) in Houston.

**Table 1:** Characteristics of 75 sampled restaurants, unweighted and weighted by number of outlets

	*n*	%, unweighted	%, weighted
Number of outlets			
< 1000	38	53	14
1000–10 000	34	47	57
> 10 000	3	4	29
Restaurant Type			
Bakery-Café	3	4	2
Beverage-Snack	9	13	18
Casual dining	9	13	4
Chicken	8	11	7
Family dining	6	8	4
LSR/Barbecue	1	1	0
LSR/Burger	13	18	24
LSR/Chinese	1	1	1
LSR/Mexican	5	7	6
LSR/Sandwich	8	11	19
LSR/Seafood	2	3	1
Pizza	10	14	14
Option to order in at least 1 city on…			
Restaurant website or app	72	96	98
At least 1 TPP	60	80	90
DoorDash	55	73	88
Grubhub	49	65	76
Uber Eats	59	79	90
Menu on at least 1 channel in…			
New York City	56	75	91
Los Angeles	60	80	93
Chicago	66	88	95
Houston	66	88	95

LSR = Limited Service Restaurant.

Calories were posted in all locations for all three foods and all three beverages on menus from forty-three of seventy-two (60 %) CRE on restaurant websites or apps, fifteen of fifty-five (27 %) on DoorDash, three of forty-nine (6 %) on Grubhub and eleven of fifty-nine (19 %) on Uber Eats. Only four CRE had calories posted for all items, in all locations in our sample, on all channels in which they participated: McDonald’s, Panera Bread, Whataburger and Jamba. There were higher location-weighted *v*. unweighted proportions of CRE with calories posted for all three foods and beverages in all locations on the restaurant website or app (75 % *v*. 60 %) and for at least one TPP (48 % *v*. 28 %) – largely because these proportions included all three of the chains with over 10 000 outlets.

Calories were posted at the point of sale in at least one location for at least one food or beverage on menus from sixty-eight of seventy-two (94 %) CRE on restaurant websites or apps, thirty-two of fifty-five (58 %) on DoorDash, six of forty-nine (12 %) on Grubhub and thirty of fifty-nine (51 %) on Uber Eats (Table 2). The four CRE for which calories were not posted at the point of sale on the restaurant website or app for any of the three foods or any of the three beverages in any location were Domino’s Pizza, Papa Murphy’s, Church’s Chicken and A&W Restaurants. Two additional CRE (Auntie Anne’s and Dickey’s Barbecue Pit) were missing calorie disclosures for all three foods and all three beverages on menus on the restaurant website or app in at least one location.

**Table 2:** Prevalence, accessibility and legibility of calorie labelling at the online point of sale on menus posted to restaurant websites and third-party ordering platforms in 2022 for the top seventy-five restaurants by number of US outlets

	Restaurant website or app				At least one third-party app				DoorDash				Grubhub				Uber Eats			
	*n*	*n*, u	%, u	%, w	*n*	*n*, u	%, u	%, w	*n*	*n*, u	%, u	%, w	*n*	*n*, u	%, u	%, w	*n*	*n*, u	%, u	%, w
Calories posted at point of sale in at least one location for…																				
at least 1 food or beverage	72	68	94 %	95 %	60	38	63 %	71 %	55	32	58 %	57 %	49	6	12 %	16 %	59	30	51 %	41 %
all 3 foods and all 3 beverages	72	56	78 %	85 %	60	25	42 %	60 %	55	22	40 %	51 %	49	4	8 %	15 %	59	17	29 %	30 %
Calories posted at point of sale in all locations for…																				
at least 1 food or beverage	72	66	92 %	94 %	60	32	53 %	61 %	55	30	55 %	54 %	49	5	10 %	13 %	59	24	41 %	32 %
all 3 foods and all 3 beverages	72	43	60 %	75 %	60	17	28 %	48 %	55	15	27 %	39 %	49	3	6 %	12 %	59	11	19 %	24 %
Calories not posted at point of sale for any of the 3 food or 3 beverage items in any location	72	4	6 %	5 %	60	22	37 %	29 %	55	23	42 %	43 %	49	43	88 %	84 %	59	29	49 %	59 %
Calories easily accessible for at least 1 food or beverage in at least 1 location	68	44	65 %	53 %	38	29	76 %	77 %	32	9	28 %	41 %	6	4	67 %	76 %	30	26	87 %	91 %
Calories fully legible for at least 1 food or beverage in at least 1 location	68	68	100 %	100 %	38	38	100 %	100 %	32	32	100 %	100 %	6	6	100 %	100 %	30	30	100 %	100 %
Mandatory disclosure regarding daily calories is present on menu in at least 1 location	72	52	72 %	84 %	60	5	8 %	13 %	55	1	2 %	9 %	49	1	2 %	10 %	59	4	7 %	5 %
Mandatory disclosures regarding availability of additional information are present on menu in at least 1 location	72	41	57 %	71 %	60	7	12 %	21 %	55	3	5 %	17 %	49	2	4 %	13 %	59	6	10 %	13 %

u = unweighted; w = weighted by # of outlets.

Mandatory disclosures state: ‘2000 calories a day is used for general nutrition advice, but calorie needs vary’ and ‘Additional nutrition information available upon request’.

Accessibility varied by channel, with calorie information for at least one food or beverage easily accessible in at least one location on menus from forty-four of sixty-eight (65 %) CRE with at least one calorie disclosure on restaurant websites or apps, nine of thirty-two (28 %) on DoorDash, four of six (67 %) on Grubhub and twenty-six of thirty (87 %) on Uber Eats. Calorie disclosures, when present, were consistently legible (100 % fully legible across all CRE in all channels).

The mandatory 2000 calorie disclosure was present on a menu in at least one location for fifty-two of seventy-two (72 %) CRE on restaurant websites or apps, one of fifty-five (2 %) on DoorDash, one of forty-nine (2 %) on Grubhub and four of fifty-nine (7 %) on Uber Eats. The mandatory Additional Information Disclosure was present on a menu in at least one location for 41 of 72 (57 %) CRE on restaurant websites or apps, three of fifty-five (5 %) on DoorDash, two of forty-nine (4 %) on Grubhub and six of fifty-six (10 %) on Uber Eats.

A chi-squared test for independence indicates that the prevalence of calorie labelling varies by channel (*P* < 0·0001). Bonferroni-adjusted comparisons indicate that the pairwise differences between each channel were highly significant (*P* < 0·0001) for all comparisons except DoorDash *v*. Uber Eats (Table 3). In particular, menus on all three TPP were significantly less likely to have calorie disclosures at the point of sale for at least one food or beverage in at least one location compared to menus on restaurant websites or apps. Menus on Grubhub were significantly less likely to have calorie disclosures than menus on DoorDash and Uber Eats.

**Table 3:** Pairwise comparisons of prevalence of calorie labelling and mandatory disclosures at the online point of sale on menus posted across channels using chi-squared tests (Bonferroni-adjusted *P*-values per Wright 1992)

**Calorie labelling**			
	Grubhub	DoorDash	Uber Eats
Restaurant Website	< 0·0001	< 0·0001	< 0·0001
Grubhub		< 0·0001	< 0·0001
DoorDash			∼1
**2000 calorie disclosure**			
	Grubhub	DoorDash	Uber Eats
Restaurant Website	< 0·0001	< 0·0001	< 0·0001
Grubhub		∼1	∼1
DoorDash			∼1
**Additional information disclosure**			
	Grubhub	DoorDash	Uber Eats
Restaurant Website	< 0·0001	< 0·0001	< 0·0001
Grubhub		∼1	∼1
DoorDash			∼1

Chi-squared tests for independence also indicate that the prevalence of the 2000 calorie disclosure and the Additional Information Disclosure vary by channel (*P* < 0·0001). Specifically for each disclosure, menus on all three TPP were significantly less likely to have the disclosure than menus on restaurant websites or apps, but there were no significant differences among the three TPP.

The likelihood of menus having calorie disclosures for at least one food or beverage on at least one TPP did not vary by city (*P* = 0·98) and was not significantly correlated with the number of outlets in a chain (Pearson’s *r* = 0·11, *P* = 0·399).

### Third-party platform websites

All three TPP websites allowed CRE to post and update menus (Table 4), with Grubhub and Uber Eats providing manual entry of menu information by CRE^(29,30)^, and DoorDash prompting restaurants to upload existing menu images for conversion to the platform (‘Attach or link your menu – we’ll do the hard part. You can send us a link or upload it. We’ll add it to your store as soon as it’s set up’^(31)^), which then could be reviewed and edited by the CRE^(32)^.

**Table 4:** Characteristics of third-party ordering platforms, 2022

	Grubhub	DoorDash	Uber Eats
Restaurants can post and update menus	Yes	Yes	Yes
Website describes how restaurants can include calorie information on menus	No	No	Yes
Website describes how restaurants can include mandatory disclosures that accompany calorie information	No	No	No
Restaurants must pay extra to include calorie information on their menus	No	No	No

We only found evidence that one platform, Uber Eats, offered instructions to restaurants explaining how to ‘Add calorie counts to an item’ and provided a dedicated ‘Energy values’ field^(33)^. Grubhub and DoorDash provided a general ‘description’ field for each menu item, but did not instruct restaurants that they could include calories in that field^(29,32)^. None of the TPP charged premiums for posting calories.

We found no evidence that any of the TPP described how restaurants could include 2000 calorie disclosures and Additional Information Disclosures on their menus.

## Discussion

Our study found that CRE are in control of their own menu information on DoorDash, Uber Eats and Grubhub, meaning under the interpretation of Federal Food, Drug, & Cosmetic Act espoused by consumer advocates, these menus would be subject to calorie labelling requirements. Despite this, twenty-two of the largest US restaurant chains (37 %) failed to include calorie counts for any menu items on any of these platforms. Only four CRE were fully compliant on all channels: McDonald’s, Panera Bread, Whataburger and Jamba.

Compliance with calorie labelling requirements was higher on the restaurant chains’ own online ordering platforms, with sixty-eight chains (94 %) posting calories for at least one food or beverage. This is consistent with previous findings that 94 % of CRE were implementing calorie labelling on their printed menus and menu boards by May 2018^(14)^. Yet only forty-three chains (60 %) consistently posted calories for all menu items on their own online platforms, and six chains (8 %) failed to include calorie counts for any online menu items in at least one location even on the chain’s own platform. For five chains (Papa Murphy’s, Church’s Chicken, A&W Restaurants, Auntie Anne’s and Dickey’s Barbecue Pit), calories did not appear during any step in the online ordering process for some or all locations on the chain’s platform. For one chain – Domino’s – calories only appeared alongside the item in the checkout ‘Cart’ after the menu item was already selected. None of the menus on this chain’s platform were therefore compliant because FDA has stated that calories ‘may not be listed on a webpage or screen that is separate from the associated menu item listed on the electronic or Internet menu’^(34)^.

The lack of compliance with labelling laws in the online restaurant food environment is consistent with previous findings of inconsistent nutrition labelling of online grocery products^(24,35)^, lack of compliance with local nutrition-related laws among US restaurant chains posting menus online^(36,37)^ and availability of nutritional information for only 20 % of menu items from major restaurant chains on Uber Eats in New Zealand^(38)^.

The variation in calorie disclosure across different locations of the same CRE suggests a lack of chain-wide guidance about including calories when posting menus online. CRE can address this by developing and disseminating guidance to their franchisees or operators.

TPP can also help by programming their platforms to better prompt CRE to enter FDA-required information. One option is to follow the approach of Uber Eats, which provides a dedicated ‘Calories’ or ‘Energy Values’ field and guidance for merchants on how to use this field. A TPP could also require this field to be filled in if a restaurant indicates it is part of a chain with twenty or more locations and is therefore a CRE. Similarly, all TPP could design their platforms to make it easier for CRE to include the mandatory 2000 calorie disclosure and Additional Information Disclosure, such as by providing this disclosure automatically on menus where the chain has indicated it is a CRE.

This study is the first to examine compliance with menu labelling requirements for online menus in the USA, but has several limitations. It was conducted during a period when FDA was not enforcing menu labelling requirements, which may increase non-compliance, but this does not explain the wide discrepancies in compliance between menus posted on TPP compared to the CRE’s own websites. We did not code the same items at all locations within each chain (just the first three foods and beverages) or the same locations within each city across TPP (since many TPP were not used by particular CRE). We did not verify that menus from the same restaurant would appear identically regardless of the location of the cell phone used for ordering because all data were collected using iPhones in the greater Boston area. However, we do not expect this would have affected our outcomes of interest because personalisation and geotargeting are not likely to be used for nutrition disclosures in the same way they may be used to tailor advertising content. It is possible that we included some items that are exempt from calorie labelling requirements because, if they appear on a menu for less than 60 d, they are exempt from mandatory disclosure^(11)^. We only assessed the presence and placement, not accuracy, of calorie disclosures. In some cases, the calorie disclosures were clearly inaccurate. For example, the Five Guys menu on the company‘s own ordering app listed a Hamburger as containing zero calories. Finally, our findings based on menus from the top seventy-five US chains may not be generalisable to smaller CRE.

### Conclusion

As consumers increasingly rely on TPP for restaurant ordering, it is critical that menus on these platforms include calories in order to promote transparency and support informed consumer decision-making. FDA should resume enforcement of menu labelling requirements and issue guidance for industry clarifying that CRE menus posted on TPP are subject to menu labelling requirements.

## Supplementary material

For supplementary material accompanying this paper visit https://doi.org/10.1017/S1368980023001799
